# Molecular Characterization of *Salmonella* Phage Wara Isolated from River Water in Brazil

**DOI:** 10.3390/microorganisms11071837

**Published:** 2023-07-19

**Authors:** Danitza Xiomara Romero-Calle, Francisnei Pedrosa-Silva, Luiz Marcelo Ribeiro Tomé, Vagner Fonseca, Raquel Guimarães Benevides, Leila Thaise Santana de Oliveira Santos, Tulio de Oliveira, Mateus Matiuzzi da Costa, Luiz Carlos Junior Alcantara, Vasco Ariston de Carvalho Azevedo, Bertram Brenig, Thiago M. Venancio, Craig Billington, Aristóteles Góes-Neto

**Affiliations:** 1 Postgraduate Program in Biotechnology, State University of Feira de Santana (UEFS), Av. Transnordestina S/N, Feira de Santana 44036-900, Brazil; xiomararomerocalle84@gmail.com (D.X.R.-C.); raquelgb@gmail.com (R.G.B.); 2 Molecular and Computational Biology of Fungi Laboratory, Department of Microbiology, Instituto de Ciências Biológicas, Universidade Federal de Minas Gerais, Belo Horizonte 31270-901, Brazil; 3 Department of Biological Sciences, Feira de Santana State University (UEFS), Feira de Santana 44036-900, Brazil; 4 Laboratory of Chemistry, Function of Proteins and Peptides, Center for Biosciences and Biotechnology, Darcy Ribeiro North Fluminense State University (UENF), Campos dos Goytacazes 28013-602, Brazil; 5 General Coordination of Public Health Laboratories/Secretariat of Health Surveillance, Ministry of Health, Brasília 70800-400, Brazil; 6 Laboratory of Cellular and Molecular Genetics, Federal University of Minas Gerais, Belo Horizonte, Minas Gerais 31270-901, Brazil; 7 KwaZulu-Natal Research Innovation and Sequencing Platform (KRISP), College of Health Sciences, University of KwaZulu Natal, Durban 4001, South Africa; deoliveira@ukzn.ac.za; 8 Department of Biological Sciences, Federal University of the São Francisco Valley, Petrolina 56304-917, Brazil; mateus.costa@univasf.edu.br; 9 Flavivirus Laboratory, Oswaldo Cruz Institute, Rio de Janeiro 21040-900, Brazil; 10 Institute of Veterinary Medicine, University of Göttingen, 37073 Göttingen, Germany; 11 Health & Environment Group, Institute of Environmental Sciences and Research, Christchurch 8540, New Zealand

**Keywords:** *Salmonella*, T5-like phage, *Salmonella* phage, whole-genome sequence

## Abstract

Antimicrobial resistance is increasing despite new treatments being employed, so novel strategies are required to ensure that bacterial infections remain treatable. Bacteriophages (phages; bacteria viruses) have the potential to be used as natural antimicrobial methods to control bacterial pathogens such as *Salmonella* spp. A *Salmonella* phage, Wara, was isolated from environmental water samples at the Subaé River Basin, Salvador de Bahia, Brazil. The basin has environmental impacts in its main watercourses arising from the dumping of domestic and industrial effluents and agricultural and anthropological activities. The phage genome sequence was determined by Oxford Nanopore Technologies (ONT) MinION and Illumina HiSeq sequencing, and assembly was carried out by Racon (MinION) and Unicycler (Illumina, Illumina + MinION). The genome was annotated and compared to other *Salmonella* phages using various bioinformatics approaches. MinION DNA sequencing combined with Racon assembly gave the best complete genome sequence. Phylogenetic analysis revealed that Wara is a member of the *Tequintavirus* genus. A lack of lysogeny genes, antimicrobial resistance, and virulence genes indicated that Wara has therapeutic and biocontrol potential against *Salmonella* species in healthcare and agriculture.

## 1. Introduction

Diarrheal disease is the second leading cause of death in children under five years of age and was responsible for the deaths of 370,000 children in 2019 [1]. *Salmonella* is one of the leading causes of this disease and more than 2600 serotypes have been identified [2]. More than 50% of serotypes belong to *S. enterica* subsp. *enterica* and they are one of the main pathogens associated with food contamination; considered to be responsible for around 94 million cases of gastrointestinal illnesses and 155,000 annual deaths worldwide [2]. In Brazil, during the period 2000–2011, *Salmonella* spp. were reported as the major cause of foodborne illness (42.27%), followed by *Staphylococcus aureus* (20.34%) and *Escherichia coli* (10.46%) [3]. The most isolated serotypes associated with non-typhoidal *Salmonella* infections worldwide are *S.* Enteriditis (65%), followed by *S. Typhimurium* (12%) and *S.* Newport (4%) [2].

The first surveillance data on resistance to antibiotics released by the WHO revealed high levels of resistance in bacterial infections in high- and low-income countries. According to the Global Antimicrobial Surveillance System [4], there was widespread occurrence of antibiotic resistance among 2.164.568 people tested for bacterial infections in 28 countries. The most reported antibiotic-resistant bacteria were *Acinetobacter* spp., *E. coli*, *Klebsiella pneumoniae*, *S. aureus*, *Streptococcus pneumoniae*, *Shigella* spp., *Neisseria gonorrhoeae*, and *Salmonella* spp. Antibiotic-resistant infections are also associated with greater morbidity and mortality, which increases healthcare costs [4]. In low-income countries, the affordability of second-line drugs and reduced access to healthcare can restrict the use of newer broad-spectrum antibiotics, and as a result, increase morbidity and mortality from antibiotic-resistant infections in these countries [5]. 

The increasing levels of antibiotic resistance in many bacterial infections have renewed interest in the exploitation of bacteriophages (phages) as therapeutic and biocontrol agents and the study of the molecular mechanisms underpinning productive infection [6,7,8,9]. Similarly, our understanding that prophages (the genomes of temperate bacteriophages either integrated into bacterial chromosomes or maintained as extrachromosomal replicons) can alter the fitness, phenotype, and global metabolism of bacteria necessitates careful identification and genomic characterization of phages. There is also significant potential for genetic engineering of phages, with applications that include transforming lysogenic phages into purely lytic phages, extending host ranges, increasing antibiotic sensitivity, and improving the lytic activity of phages via removal of repressor genes [10,11].

The identification of the life cycle of phages and their virulence and antimicrobial resistance genes through genomic characterization is essential for the application of phage therapy and biological control. Whole-genome sequencing (WGS) has proven very useful in foodborne outbreak investigations and phage identification. Illumina short-read sequencing technology has proven to be robust for characterizing pathogens of clinical care [12], but it is unable to resolve repetitive and GC-rich regions, thus producing unresolvable regions in the underlying genome assembly [13]. In comparison to the Illumina sequencing platform, there are also relatively few reports of phages sequenced by single-molecule real-time (SMRT) technologies such as those provided by Oxford Nanopore Technologies (ONT) or PacBio [9]. We are not aware of any studies that have tested hybrid genome assembly, which combines short-read Illumina and SMRT sequencing data, for *Salmonella* phages, so this is an aspect we wished to explore.

Phages have been used for the biocontrol of food-borne pathogens, such as *Salmonella* in diverse food matrices [12,13,14,15,16,17]. Also, commercially available phage products such as SalmoFresh, Armament, and Salmonelex have been used for the biocontrol of *Salmonella* in foods [12,18]. Since bacteria and phages have co-evolved for billions of years, bacteria have developed multiple defense systems against phages [19]. Therefore, to ensure continued biocontrol efficacy, an ongoing search and identification of new candidate phages with high lytic activity is warranted.

Considering the great potential of phages as antimicrobial agents in *Salmonella* biocontrol, this study was undertaken to isolate phages from the Subaé River in Brazil able to infect *S. enterica*. The Subaé River was previously characterized as highly polluted, including the presence of *Salmonella*, so the probability of finding *Salmonella* phages was thought to be higher than other regions [20]. Phenotypic and genetic analyses were used to characterize one of the *Salmonella* phages identified with the aim of testing its safety and suitability for further development as a biocontrol agent for this key pathogen of interest for healthcare in Brazil. We also aimed to see if hybrid genomic sequencing provided benefits in assembling phage genomes for subsequent safety analyses.

## 2. Materials and Methods

### 2.1. Isolation and Purification of Salmonella Phages

Environmental water samples were obtained from the Subaé River Basin, Salvador de Bahia, Brazil. The basin has environmental impacts in its main watercourses arising from the dumping of domestic and industrial effluents and agricultural and anthropological activities [20]. Three samples of water (approximately 100 mL) were collected from the Subaé River, Santo Amaro city, Salvador de Bahia, Brazil (12°31′46.77″ S 38°44′1.24″ W).

The samples were transported in a refrigerated box (4–8 °C) to a laboratory where phage purification was undertaken immediately.

The water samples (1 mL) were each added to 10 mL of phage buffer (10 mM Tris HCl (pH 7.5), 68.5 mM NaCl, 10 mM MgSO_4_ and 5 mM CaCl_2_), mixed and incubated for 10 min at room temperature. The suspensions were filtered (0.22 µm, Kasvi, São José dos Pinhais, Brazil). For enrichment, 2.5 mL of the filtered sample, 2.5 mL of log-phase *Salmonella* Typhimurium ATCC 14028, and 10 mL of LB culture media (Kasvi) were mixed and incubated for 18 h at 37 °C. The samples were centrifuged at 3500 rpm for 10 min and the supernatant was filtered (0.22 µm, Kavsi). The filtrate (100 µL) was mixed with 100 µL log-phase *Salmonella* Typhimurium ATCC 14028 and 10 mM of CaCl_2_ (Dinámica, São Paulo, Brazil), incubated at room temperature for 10 min, added to an LB top-agar overlay plate, and incubated overnight at 37 °C. A single clear plaque was selected and propagated on the host five times to ensure virulence and purity. Phage buffer (5 mL) was added to each of the 5 confluent lysis agar plates, and the buffer was recovered and centrifuged at 3500 rpm for 10 min and filtered (0.22 µm, Kavsi). The filtered suspensions were ultra-centrifuged at 100,000× *g* for 1.5 h, and the supernatant was discarded, 500 µL of SM buffer (50 mM Tris–HCl (pH 7.5), 100 mM NaCl, 8.1 mM MgSO_4_, and 0.01% (*w*/*v*) gelatin) was added to the pellet and stored at 4 °C [21]. 

### 2.2. Preparation of High Titer Phage Stocks

The purified phages were diluted serially in SM buffer to give near-confluent lysis of the host on the LB top-agar overlay plates. SM buffer (5 mL) was added to each plate and left at room temperature for at least 1 h, and the plates were swirled regularly. The buffer was decanted and vortex-mixed briefly before shaking for 30 min. The solution was centrifuged at 11,000× *g* for 10 min, and 5.8% NaCl was added and incubated at 37 °C for 1 h [22]. Then, 10% PEG 6000 was added and incubated overnight at 4 °C. The supernatant was removed by centrifugation at 15,000× *g* for 1 h and the pellet was suspended in LB. 

### 2.3. Phage Host Range 

The host range of the isolated phages was determined by challenging them against 34 strains of *bacteria Salmonella* (Table 1). Exponential growth phase suspensions of the host strains were prepared (OD_600_ = 0.7). LB top-agar overlays were inoculated with 100 μL of the host and poured on an LB agar base plate previously marked in a grid to allow for identification of each inoculum [22]. After the solidification of the soft agar, 5 μL drops of phage from a 2 × 10^3^ PFU/mL suspension (~10 PFU/spot) were placed in triplicate on double-layered agar LB plates containing each individual host strain [22,23]. Infectivity was scored positive only when individual plaques were present.

### 2.4. DNA Isolation

To isolate the phage DNA, 2 µL of 1 mg/mL DNAse (Sigma-Aldrich, Burlington, MO, USA) and 20 µL of 1 mg/mL RNAse (ThermoFisher Scientific, Waltham, MA, USA) were added to 2 mL of filtered culture supernatant for 30 min at 37 °C. Then, 40 µL of 2 M ZnCl_2_ (Dinámica) was added and the suspension was incubated for 5 min at 37 °C and centrifuged at 12,000× *g* for 1 min. The supernatant was discarded, and 1 mL of TES (0.1 M Tris-HCl pH 8 (Sigma-Aldrich, SAO, Brazil), 0.1 M EDTA (Promega, Madison, WI, USA), 0.3% SDS (Promega) was added to the pellet and incubated for 15 min at 60 °C. Proteinase K (40 µL, 20 mg/mL (ThermoFisher Scientific) was added and incubated for 90 min at 37 °C. Subsequently, 1.5 mL of 100% ethanol and 6 M Guanidine-HCl was added to the 1 mL sample. A QIAamp MinElute Virus Kit (Qiagen, Hilden, Germany) columns were used for DNA isolation and purification.

### 2.5. Whole-Genome Sequencing by MinION

Nanopore WGS sequencing was carried out at the Molecular and Computational Biology of Fungi Laboratory, Federal University of Minas Gerais (UFMG). The DNA library was prepared with a ligation sequencing kit (SQKRAD004, Oxford Nanopore Technologies, Oxford, UK) according to the manufacturer’s instructions. Libraries were sequenced with qualified FLO-MIN106 flow cells for 16 h on a MinION (Oxford Nanopore Technologies) [24,25].

The quality of the sequencing was verified through the FastQC v0.11.9 program (https://github.com/s-andrews/FastQC, accessed on 15 January 2022). The Porechop v0.2.4 program (https://github.com/rrwick/Porechop, accessed on 15 January 2022) was used for the detection and elimination of the adapters and the demultiplexing of the Nanopore reads. Canu v2.1.1 was used to monitor and correct possible sequencing errors [26]. The de novo assembly, based on de Bruijn graphs, of the corrected readings was performed using Flye v2.8.3 [27]. Contigs were polished with Racon v1.4.22 [28] which previously took the reads mapped by BWA v0.7.17 [29].

### 2.6. Whole-Genome Sequencing by Illumina

The library was prepared using 1 µg of purified phage DNA with the NEBNext Fast DNA Fragmentation and Library Preparation Kit (New England Biolabs, MA, USA) following the manufacturer’s recommendations. The library quality was assessed using Agilent 2100 Bioanalyzer equipment, and paired-end DNA sequencing was carried out on an Illumina HiSeq 2500 platform. After sequencing, the raw read quality was assessed using FastQC v0.11.5 (https://github.com/s-andrews/FastQC, accessed on 15 January 2022). The reads were assembled using SPAdes v3.15.3 [30]. Hybrid assemblies using [26] both Illumina and MinION reads [31] were performed using Unicycler Genome quality, and the completeness for each assembly was evaluated using QUAST v4.6.0 [32].

### 2.7. Genome Annotation and Analysis

Automated annotation of genes was performed via Prokka v1.14.6 [33] followed by manual curation. The completeness of the phage genome sequences was tested using checkV [34]. Preliminary identification of the closest relatives of the Wara phage was completed using PhageClouds (http://phagecompass.dk/, accessed on 2 January 2023). PhageLeads was used to assist in the prediction of therapeutic suitability (http://phagecompass.dk/, accessed on 2 January 2023). Abricate was used to identify antimicrobial resistance and virulence genes [35]. Genomic comparison of the Wara phage genome was performed using the reference of *Salmonella* phage s131 (NC_048009.1) using BRIG (http://brig.sourceforge.net/ (accessed on 2 January 2023)) [36] and CorelDRAW (https://www.coreldraw.com/la/ (accessed on 2 January 2023)).

### 2.8. Proteome-Based Clustering and Phylogenetic Analysis

To investigate the phage taxonomy assignment of *Salmonella* phage Wara, we performed a shared proteome clustering analysis using the vConTACT2 tool [37]. The resulting network graph was visualized and annotated with Gephi [38]. Further, we downloaded 71 genomes of the *Markadamsvirinae* subfamily from the NCBI RefSeq database on December 2022. Comparative genome analysis of the *Markadamsvirinae* subfamily was performed by annotating the RefSeq genomes with Prokka [33], followed by analysis with Roary v.3.6, using an identity threshold of 80% to determine the gene clusters [39]. The core genes were aligned with MAFFT v.7.394 [40], and a maximum-likelihood phylogenetic tree was inferred with IQ-tree v.1.6.12 [41], using the best-fitting model GTR + F + I + G4. The bootstrap support was evaluated using the ultra-fast bootstrap method with 1000 replicates. The resulting phylogenetic tree was visualized and modified using iTOL v4 [42]. All-against-all average nucleotide identity based on MUMmer alignment (ANIm) was performed with pyani v.0.27 [43].

### 2.9. Electron Microscopy

The high titer phage stocks were prepared in LB media (see Section 2.2), and the solutions were placed on electron microscopy (EM) grids and stained with 1% phosphotungstic acid (Vetec, Bs. As., AR). Micrographs were taken at 135,000× at 120 kV using a Tecnai G2-12-FEI Spirit Biotwin EM instrument [22] at the microscopy center at UFMG.

## 3. Results

### 3.1. Isolation of Salmonella Phages

**Figure 1:**
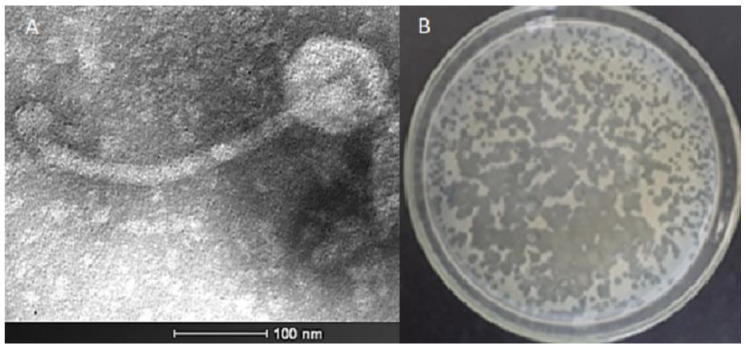
(**A**) Electron micrograph and (**B**) top-agar plate of Salmonella phage Wara.

Samples of water from the Subaé River located in Santo Amaro, Brazil, were analyzed for the presence of phages able to infect *Salmonella enterica* sv. Typhimurium using enrichment and top-agar overlay techniques. After the isolation and purification of many candidate phages, one phage was identified for further study as it consistently produced clear plaques; this phage was named *Salmonella* phage Wara (Figure 1).

### 3.2. Electron Microscopy of Wara

Structural analysis of Wara was undertaken using transmission electronic microscopy (TEM). The average dimensions of the phage Wara were a capsid diameter of 90.28 nm and a tail length of 274.31 nm (Figure 1). The Wara capsid was of icosahedral type and the tail appeared flexible, and these morphological features and dimensions corresponded to the Siphovirus T5-type [4].

### 3.3. Wara Host Range

To determine the host range of Wara, 34 local and overseas strains of *S. enterica* of some of the key serotypes of concern for Brazil were tested for infectivity using a top-agar assay. Wara showed activity against nine of these strains, including infectivity on *Salmonella* serovars Enteritidis, Typhimurium, and Minnesota but not Heidelberg. (Table 1). Efficiency of plating on the susceptible strains did not indicate any correlation to serovar and was in a narrow range (0.88–1.29, relative to strain 14028).

### 3.4. Wara Whole-Genome Sequencing and Assembly

The genome of Wara was analyzed using two next-generation sequencing platforms: ONT MinION and Illumina HiSeq. Two platforms were used as we wished to compare the effectiveness of these different approaches for sequencing the phages. After pre-processing, the number of reads from HiSeq was 40,000 and the number of reads from MinION was 4000. The sequencing depth for HiSeq was >100× and for MinION, it was 30×. The number of reads was 2,829,936 bp for Illumina HiSeq and 22,541 bp for MinION.

Three assembly strategies were tested for the sequence data from the two platforms: (1) SPAdes assembly on HiSeq data, (2) Racon assembly on MinION data, and (3) Unicycler hybrid assembly on both HiSeq and MinION data (Table 2). QUAST evaluations of the genome assemblies’ suggested MinION with Racon assembly showed the best performance, producing 1 contig with a total length of 112,042 bp and N50 of 112,042 bp (Table 2). According to the BLAST (https://blast.ncbi.nlm.nih.gov/Blast.cgi, accessed on 15 January 2022) analysis, for 92% of the query cover, a 0 E-value was reported for the Wara phage using the three assemblies, 94.48% of identity using MinION, 96.37% of identity using HiSeq assembly, and 96.36% of identity using hybrid assembly to the same phage, *Salmonella* phage S131 (NC_048009.1).

### 3.5. Wara Genome Annotation and Analysis

**Figure 2:**
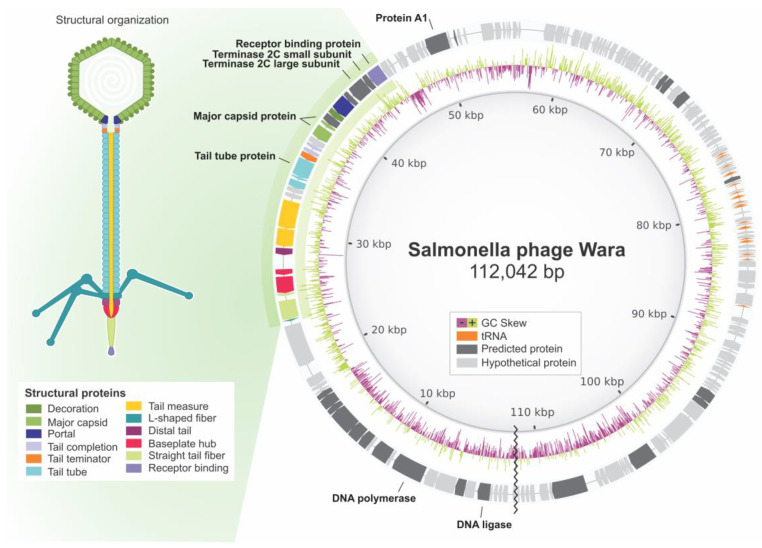
Genome organization and structural representation of *Salmonella* phage Wara based upon annotated genes and predicted encoded proteins. Shades of gray indicate nucleotide similarity to *Salmonella* phage S131 (darker is higher). Image made with BRIG and CorelDraw.

Annotation of the three Wara phage genome assemblies and the reference phage genome S131 was undertaken using Prokka with the Caudovirales database (Table 3). The Racon assembly of the MinION data identified the highest number of coding sequences (CDS; 170), but all three assembly methods produced the same number of tRNAs (21). These results and the assembly statistics led us to undertake the phage Wara genome assembly using Racon on the MinION reads to compare with the reference genome S131. Genes encoding the major phage proteins such as the terminase, major capsid, receptor b, tail tube, DNA polymerase, DNA ligase, and protein A1 were identified, along with hypothetical unknown proteins, and the genome structure compared with *Salmonella* phage S131 (Figure 2).

To determine genome completeness, CheckV was applied to the three assemblies. The results showed high quality: a lower completeness of 88.16% and an upper completeness of 100%, no host contamination was detected, and 137 genes were predicted. PhageLeads analysis of the Wara genome did not find any predicted temperate lifestyle genes. Antimicrobial resistance and virulence genes were searched in the Wara genome using Abricate, but no genes of these types were detected. PhageClouds analysis of the Wara genome suggested that Phage_NBSal003 was the closest nucleotide match, with a distance of 0.0376.

### 3.6. Proteome-Based Clustering and Phylogenetic Analysis of Wara

**Figure 3:**
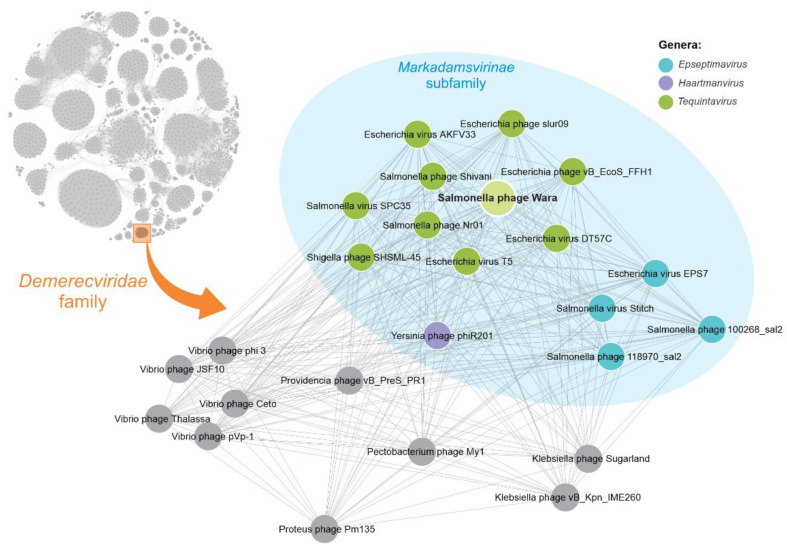
Proteome-based similarity network for Wara.

**Figure 4:**
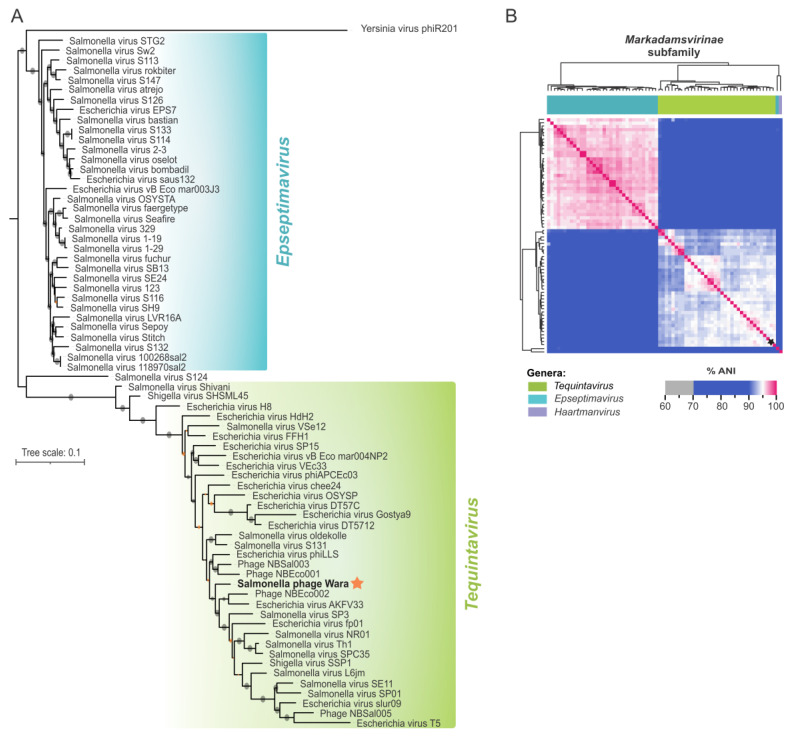
Phylogeny analysis and genomic diversity of the *Markadamsvirinae* subfamily. (**A**) Phylogenetic tree of *Markadamsvirinae* genomes highlighting two phylogenetic clades that correspond to the genera *Epseptmavirus* and *Tequintavirus*. The *Salmonella* phage Wara genome is in bold and highlighted with an orange star. A total of 46 core genes were used to build a maximum-likelihood phylogenetic tree using IQ-tree (see methods for details). Bootstrap values below and above 70% are represented by orange and black dots, respectively. (**B**) Pairwise average nucleotide identity (ANI) was calculated with 72 *Markadamsvirinae* genomes. Colors depict the degree of genome identity. The *Salmonella* phage Wara genome is highlighted with a black star.

Proteome clustering and network analysis using vConTACT2 [37] assigned the Wara genome to a viral cluster with 24 known phages, all of them currently classified as Demerecviridae (Figure 3). To further refine the phylogeny, we compared the Wara genome with members of the Markadamsvirinae family, and we downloaded all these genomes from the RefSeq database (n = 71, December 2022). The core genome (genes present in at least 96% of the genomes) of the 72 Markadamsvirinae genomes was determined using Roary [39] comprising a total of 46 core genes. To infer evolutionary relationships between the Wara isolate and the RefSeq genomes, we performed a maximum-likelihood phylogenetic reconstruction using the 46 core genes. Our results showed that *Salmonella* phage Wara belongs to the monophyletic clade containing 34 phages of Tequintavirus (Figure 4A), representing a new member of this genus. These results are supported by ANI analysis, which showed a pairwise genomic identity above 90% of the Wara phage with other Tequintavirus genomes (Figure 4B).

The predicted proteome of *Salmonella* phage Wara was clustered with the proteome of 24 phages of the *Demerecviridae* family. Phages belonging to the *Markadamsvirinae* subfamily are shaded in blue. Each node represents a single phage genome, and the edge represents significant similarity between proteomes of connected phages.

## 4. Discussion

The Subáe River frequently has livestock in and around it and appears to have high loads of organic waste. Recently, our group has described the isolation of *Salmonella* from the banks of this waterway [8]. In this study, a *Salmonella* phage, Wara, was isolated from the water of the river and the phage produced clear plaques on top-agar overlay plates which suggested that the phage was virulent rather than temperate. Visualization of the phage structure by TEM suggested that Wara was a Siphovirus of the T5 type [44]. T5 phages typically consist of an icosahedral capsid containing a large molecule of double-stranded DNA (dsDNA) (121.75 kbp) attached to a long flexible noncontractile tail [45], which is very similar to the morphological and molecular data we obtained for Wara.

The host range is an important property to determine for phages, particularly if they are to be used for biocontrol. The *Salmonella* phage Wara infected several serotypes of *Salmonella* such as Enteritidis, Typhi, and Typhimurium and only a few types of Minnesota, with no Heidelberg serovars infected. Similarly, Ref. [46] reported that *Salmonella* phages Salmacey-1, Salmacey-2, and Salmacey-3 had lytic activity on multiple serovars including Typhimurium, Enteritidis, Kentucky, and Typhi. It is likely that the receptor genes identified, receptor b and the tail protein, are responsible for the host specificity of the Wara phage. Further study of these receptors and detailed investigations of phage kinetics on these hosts would be valuable for increasing the knowledge of Wara phage–host interactions, the ecology and evolution of phages in general, and their application in health and veterinary care.

However, phage host range is not always genera restricted, and the selection of subpopulations of phages capable of amplification in alternative genera may provide a tool for the selection of broad-hostrange phages for the pathogen of interest [47]. The selection of non-pathogenic host isolates to support the replication of *Salmonella* bacteriophages may also allow improved safety for phage applications to farmed animals because this would reduce the purification requirements of the phages(s) [47]. Another study showed that the E4 phage can infect different *Salmonella enterica* serovars, including all tested motile serovars and non-motile *Salmonella* pullorum [48]. It could not infect other Gram-negative and Gram-positive bacteria, comprising lactobacilli, which are part of the normal flora [48]. Even though host specificity occasionally restricts their practical application, it is still considered one of the significant advantages of using phages as this causes the balance of microflora not to be disturbed during treatment, which in turn prevents secondary infections and accelerates the treatment process [49]. Nonetheless, broad-host-range phages are, on the other hand, regarded as valuable tools in some uses. In respect to phage therapy, a broad-host-range phage that eliminates various bacterial pathogen species would be analogous to a broad-spectrum antibiotic [50]. According to these features, Wara can be a candidate for biological control and phage therapy.

The genome sequence of Wara was determined using both MinION and Illumina technologies, either alone or in combination (hybrid assembly). The sequencing and assembly quality data indicated that MinION data alone, with Racon assembly, gave the best results and identified the most coding sequences. Although, no major differences were detected between the different assemblies and the reference *Salmonella* phage S131 [51]. However, it could be that the pipeline used for hybrid assembly in this study needs to be improved because this pipeline is optimized for bacteria and not phages. In comparison to Illumina sequencing, there are relatively few reports of phages sequenced solely by MinION or other single-molecule sequencing technologies such as PacBio. However, both MinION and PacBio were successfully applied for the detection of phages with modified nucleotides or phages shown to be refractory to conventional sequencing approaches [9].

Phage genome analysis is essential in order to identify potential toxin-encoding, antibiotic, and virulence genes before further characterization and development of phages for biocontrol or phage therapy. Therefore, the use of the best sequencing, assembly, and annotation tools is vital for this process.

Most of the protein-coding sequences identified in the Wara genome were hypothetical proteins. Rivera et al. [52] described *Salmonella* phage STGO-35-1, and Wara-like receptor-binding proteins and tail tube proteins were identified, along with structural protein major capsid and DNA polymerase. A *Salmonella* phage, SSBI34, was reported by Sattar et al., [53] and like our study, it encoded a DNA ligase and DNA polymerase I and III, indicating independence from host polymerases for DNA replication. The A1-protein-encoding gene was detected in Wara, which is involved in the degradation of host DNA and the shutoff of host genes and phage pre-early genes (https://www.uniprot.org/uniprotkb/Q6QGT3/entry, accessed on 15 January 2022).

Phylogenetic and network analysis showed *Salmonella* phage Wara belonged to the Markadamsvirinae subfamily, Tequintavirus genus. Moreover, proteome analysis revealed the Wara genome had similarity with phages belonging to the Markadamsvirinae subfamily associated with bacterial hosts in the enteric genera *Salmonella*, *Escherichia*, *Shigella*, and *Yersinia*.

A genomic biosafety analysis indicated that the Wara phage did not have a lysogenic cycle, and no antimicrobial resistance or bacterial virulence genes were detected. Therefore, the Wara phage has the potential to be considered for use in phage therapy and biological control applications for *Salmonella* and potentially other species.

## 5. Conclusions

We isolated and characterized a *Salmonella* phage from the water of the Subáe River in Santo Amaro, Brazil. TEM and genome analysis indicated that Wara was a member of the Tequintavirus genus. We demonstrated that MinION sequencing and Racon assembly were the best tools for the assembly of the genome sequence. The lack of lysogeny genes, antimicrobial resistance. and virulence genes indicated that Wara has therapeutic and biocontrol potential against *Salmonella* species in healthcare and agriculture.

## Figures and Tables

**Table 1:** Host range of Wara.

*Salmonella enterica* Serovar	Strain	EOP (cf. 14028)	Source */Reference
Enteritidis	ATCC 13076	0.88	LCMG/UFMG
Enteritidis	SE3	1.12	DBS/UEFS
Enteritidis	SE4	0.88	DBS/UEFS
Heidelberg	SH1	-	DBS/UNIVASF
Heidelberg	SH10	-	DBS/UNIVASF
Heidelberg	SH2	-	DBS/UNIVASF
Heidelberg	SH3	-	DBS/UNIVASF
Heidelberg	SH4	-	DBS/UNIVASF
Heidelberg	SH5	-	DBS/UNIVASF
Heidelberg	SH6	-	DBS/UNIVASF
Heidelberg	SH7	-	DBS/UNIVASF
Heidelberg	SH8	-	DBS/UNIVASF
Heidelberg	SH9	-	DBS/UNIVASF
Minnesota	SM1	1.29	DBS/UNIVASF
Minnesota	SM10	0.88	DBS/UNIVASF
Minnesota	SM2	-	DBS/UNIVASF
Minnesota	SM3	-	DBS/UNIVASF
Minnesota	SM4	-	DBS/UNIVASF
Minnesota	SM5	-	DBS/UNIVASF
Minnesota	SM6	-	DBS/UNIVASF
Minnesota	SM7	-	DBS/UNIVASF
Minnesota	SM8	-	DBS/UNIVASF
Minnesota	SM9	-	DBS/UNIVASF
Typhi	I	-	LCMG/UFMG
Typhi	Ia	1.18	LCMG/UFMG
Typhi	II	-	LCMG/UFMG
Typhi	III	-	LCMG/UFMG
Typhi	IV	-	LCMG/UFMG
Typhimurium	14028	1.00	ATCC
Typhimurium	14088	0.88	ATCC
Typhimurium	II	1.00	LCMG/UFMG
Typhimurium	III	1.29	LCMG/UFMG
Typhimurium	IV	-	LCMG/UFMG

* LCMG (Laboratory of Cellular and Molecular Genetics), UFMG (Universidade Federal de Minas Gerais), ATCC (American Type Culture Collection), DBS (Department of Biological Sciences), UNIVASF (Universidade Federal do Vale do São Francisco), and UEFS (State University of Feira de Santana).

**Table 2:** Summary statistics for the assembled genome of Wara.

Assembly Method	Racon	SPAdes	Unicycler	
Sequencing Platform	MinION	HiSeq	MinION/HiSeq	Reference NC_048009.1
Number of contigs ≥ 0 bp	1	112	1	1
Number of contigs ≥ 50,000 bp	1	1	1	1
Largest contigs (bp)	112,042	110,012	110,012	110,091
Total length ≥ 50,000 bp	112,042	110,012	110,012	110,091
GC (%)	39.15	39.17	39.17	39.22
N50	112,042	110,012	110,012	110,091
L50	1	1	1	1

**Table 3:** Prokka annotation analyses using the Caudovirales database.

Feature	Reference NC_048009.1	Racon/ MinION	SPAdes/ HiSeq	Unicycler/ HiSeq + MinION
CDS	155	170	161	156
tRNA	23	21	21	21

## Data Availability

Not applicable.
